# Supplementary material for: Sex-Specific and Long-Term Impacts of Early-Life Venlafaxine Exposure in Zebrafish
Source: Biology (Basel). 2022 Feb 6;11(2):250. doi: 10.3390/biology11020250 (PMC8869491; doi:10.3390/biology11020250)
Supplement: Supplementary file 1 [file biology-11-00250-s001.zip › biology-1559793-supplementary/biology-1559793-supplementary - proof - corrected vj final.pdf]

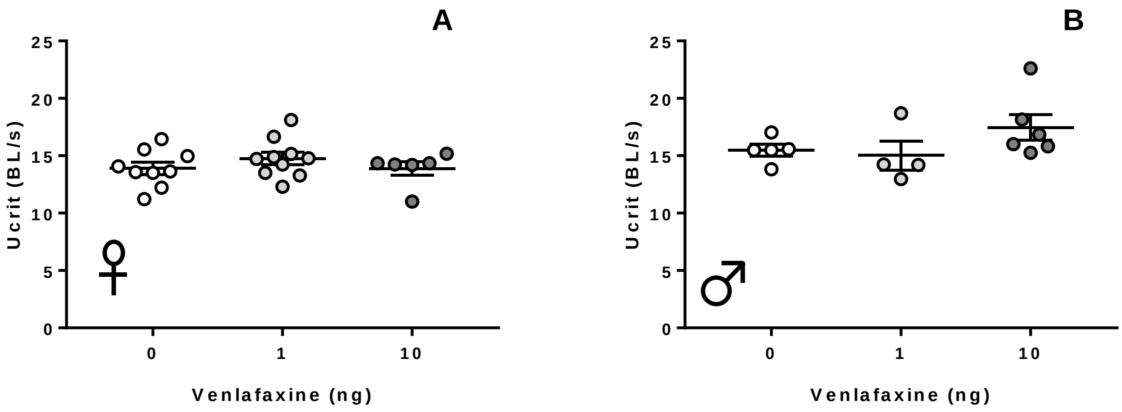

Figure S1. Swimming performance of adult zebrafish. Critical swimming speed (Ucrit) of 10–12-month-old female (**A**) and male (**B**) zebrafish exposed to either 0, 1, or 10 ng venlafaxine at the 1–4 cell stage (n = 4–10 for swimming performance). Different letters denote significant differences between groups. Individual data points and mean are shown for each graph (± S.E.M).

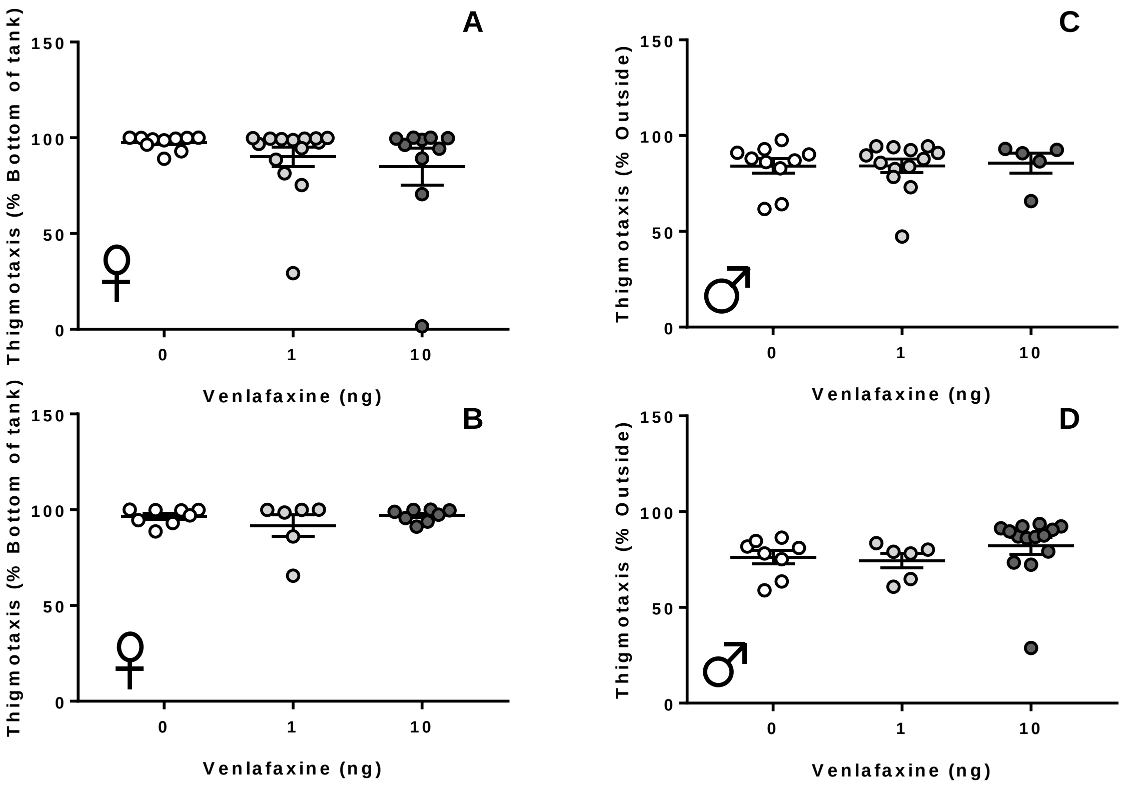

Figure S2. Thigmotaxis in adult zebrafish. Thigmotaxis of the vertical assessment (**A** and **B**), and open field assessment (**C** and **D**) of female (**A** and **C**) and male (**B** and **D**) adult zebrafish exposed to either 0, 1, or 10 ng venlafaxine at the 1–4 cell stage (n = 4–10 for swimming performance). Individual data points and means are shown for each graph (±S.E.M).
